# Ultra high-field (7 T) multi-resolution fMRI data for orientation decoding in visual cortex

**DOI:** 10.1016/j.dib.2017.05.014

**Published:** 2017-05-24

**Authors:** Ayan Sengupta, Renat Yakupov, Oliver Speck, Stefan Pollmann, Michael Hanke

**Affiliations:** a Department of Experimental Psychology, Institute of Psychology II, Otto-von-Guericke University, Universitätsplatz 2, 39016 Magdeburg, Germany; b Department of Biomedical Magnetic Resonance, Institute for Experimental Physics, Otto-von-Guericke University, Leipziger Str. 44, 39120 Magdeburg, Germany; c Center for Behavioral Brain Sciences, Universitätsplatz 2, 39016 Magdeburg, Germany; d Leibniz Institute for Neurobiology, Brenneckestr. 6, 39118 Magdeburg, Germany; e German Center for Neurodegenerative Disease (DZNE), site Magdeburg, Leipziger Straße 44, Germany; f Psychoinformatics lab, Institute of Psychology II, Otto-von-Guericke University, Universitätsplatz 2, 39016 Magdeburg, Germany; g Sir Peter Mansfield Imaging Centre, School of Physics & Astronomy, University of Nottingham, UK

## Abstract

Multivariate pattern classification methods have been successfully applied to decode orientation of visual grating stimuli from BOLD fMRI activity recorded in human visual cortex (Kamitani and Tong, 2005; Haynes and Rees, 2005) [12], [10]. Though there has been extensive research investigating the true spatial scale of the orientation specific signals (Op de Beeck, 2010; Swisher et al., 2010; Alink et al., 2013; Freeman et al., 2011, 2013) [2], [15], [1], [4], [5], it remained inconclusive what spatial acquisition resolution is required, or is optimal, for decoding analyses. The research article entitled “The effect of acquisition resolution on orientation decoding from V1 BOLD fMRI at 7 T” Sengupta et al. (2017) [14] studied the effect of spatial acquisition resolution and also analyzed the strength and spatial scale of orientation discriminating signals. In this article, for the first time, we present empirical ultra high-field fMRI data, obtained as a part of the aforementioned study, which were recorded at four spatial resolutions (0.8 mm, 1.4 mm, 2 mm, and 3 mm isotropic voxel size) for orientation decoding in visual cortex. The dataset is compliant with the *BIDS* (Brain Imaging Data Structure) format, and freely available from the *OpenfMRI* portal (dataset accession number: http://openfmri.org/dataset/ds000113c ds000113c).

**Specifications Table**

**Table:** 

Subject area	Neuroimaging
More specific subject area	Early visual system
Type of data	Ultra High Field (7 T) BOLD fMRI
How data was acquired	Siemens Magnetom 7 T whole body MRI scanner (Erlangen, Germany) with a 32 receive channel head coil (Nova Medical, Wilmington, MA)
Data format	Raw and distortion corrected BOLD fMRI data stored in compressed NIFTI format; BIDS-compliant
Experimental factors	Acquisition resolution (within-subject factor; 0.8 mm, 1.4 mm, 2 mm, and 3 mm isotropic voxel size)
Experimental features	BOLD fMRI data acquired from 7 participants while they fixated oriented flickering gratings
Data source location	Magdeburg, Germany
Data accessibility	Data available at *OpenfMRI* portal (dataset accession number: http://openfmri.org/dataset/ds000113c ds000113c), as well as Github/ZENODO (DOI: doi:10.5281/zenodo.46756)

**Value of the data**

• First publicly available dataset to provide ultra high-field, multi-resolution BOLD fMRI data for a uniform stimulation paradigm targeting the representation of visual orientations in early visual cortex.
• Extension of the 〈http://studyforrest.org〉 dataset with a large amount of auxiliary data for all scanned participants, such as T1, T2, and SWI data for tissue segmentation and blood vessel locations, as well as several additional visual and auditory stimulation paradigms including a retinotopic mapping, and two natural movie stimuli.
• Compliant with the brain imaging data structure (BIDS) standard, hence highly suitable for automated processing.
• Potent dataset for optimization and benchmarking of algorithms, such as pattern classification and feature extraction.
• Flexible and unrestricted data access down to the level of individual files facilitate cloud-based analysis and utilization in (web-based) demonstrations.

## 1 Data

The dataset of this article was being collected as a part of [14], in which the effect of acquisition resolution on orientation decoding from primary visual cortex and the strength and the true spatial scale of decoding signals were analyzed. This dataset is published publicly in compliance with Brain Imaging Data Structure (BIDS) specification [6]. All participants recruited in this study previously participated in *the studyforrest project* ([8], [9], [7]. The T1, T2, SWI structural images and the fMRI dataset for retinotopic mapping, which are available from [13], have also been utilized in this study. This section provides information about the released data, but limits its description to aspects that extends the BIDS specifications. All files related to the data acquisitions for a particular participant (described in Table 1) can be located in a sub-<ID>/ses-r<RES>/ directory, where ID is the numeric subject code, and RES is a two-digit acquisition resolution identifier.

**Table 1:** Description of the files in the dataset containing acquired data and associated meta-data for a particular participant.

Data files	Description
participants.tsv	Basic demographics for each participant: gender, age group (five-year bin size), and self-reported handedness.
*run-??_bold.nii.gz	raw fMRI BOLD images.
*rec-dico_run-??_bold.nii.gz	Distortion corrected BOLD images [11].
*motion_physio.tsv.gz	Whitespace-delimited 6-column text file with motion estimates with translation along X, Y, Z axes in mm and rotation around X, Y, Z axes in deg. It contains one row per fMRI dynamic for each acquisition run.
*_events.tsv	Text files, one for each acquisition run, with 4-columns. The columns describe the onset, duration of a stimulus trial (in seconds) and the associated stimulus orientation (in deg) presented in the left (lh_orientation), and in the right hemifield (rh_orientation). A stimulus orientation label of none indicates that no stimulus was present in the respective trial (unilateral stimulation).
*task-coverage*	fMRI acquisition with enhanced spatial coverage at 0.8 mm resolution to facilitate alignment of high-resolution BOLD images with limited coverage to other functional or structural images.

In order to anonymize the data, information on center-specific study and subject codes have been removed using an automated procedure described in [8]. All human participants were given integer IDs that are consistent across all other data releases of the *studyforrest* project [8].

## 2 Experimental design, materials and methods

Functional MRI data were recorded from seven healthy volunteers (age 21–38 years, 5 males) with normal or corrected to normal vision. The experiment had an event related design with each trial consisting of 3 s of flickering grating display and 5 s of medium gray. The random phase shifted sine wave gratings (0.8–7.6° eccentricity, 100% contrast, spatial frequency of 1.4 cycles per degree of visual angle, 125 ms ON/OFF period) were independently oriented at either 0°, 45°, 90°, or 135°. There were 30 such trials in an experimental run. For every session 10 runs were acquired. For additional details on the procedures please refer to [14].

### 2.1 Functional imaging

The participants were scanned using a 7 T whole body scanner (Siemens, Erlangen, Germany) and a 32 receive channel head coil (Nova Medical, Wilmington, MA). T2*-weighted echo planar images (EPI) (TR/TE=2000/22 ms, FA=90°) with the coverage of the primary visual cortex (parallel to the calcarine sulcus on a tilted axial plane), were acquired for 4 different spatial resolutions in individual scanning sessions. In each scanning session 10 separate scans (one for each experimental run consisting of 121 dynamics) were performed on each subject. In order to position the field-of-view to the same volume in each scan for each subject, *Siemens AutoAlign Head LS* system was used [3]. The data acquired in all scans were distortion corrected ([11]). The functional acquisition parameters used in this experiment are described in Table 2.

**Table 2:** fMRI acquisition parameters used in this experiment. All EPI scans implemented ascending slice acquisition order and used a 10% inter-slice gap to minimize cross-slice excitation.

Acquisition resolution	Field of view (FoV)	Acquisition matrix	GRAPPA accel. factor	Phase encoding
0.8 mm iso	128×166.4 mm (AP×LR)	160×208, 32 slices	4	L–R
1.4 mm iso	196 mm	140×140, 32 slices	3	A–P
2.0 mm iso	200 mm	100×100, 37 slices	2	A–P
3.0 mm iso	198 mm	66×66, 37 slices	2	A–P

The 0.8 mm acquisition had a small coverage and in order to aid corresponding co-registration with the structural image, an additional EPI acquisition was performed that used the same auto-alignment procedure. The details of the acquisition and the co-registration procedure is described in [14].
