# Supplementary material for: Ultra high-field (7 T) multi-resolution fMRI data for orientation decoding in visual cortex
Source: Data Brief. 2017 May 24;13:219–22. doi: 10.1016/j.dib.2017.05.014 (PMC5459569; doi:10.1016/j.dib.2017.05.014)
Supplement: Supplementary file 1 — Supplementary material [file mmc1.pdf]

Conflict of interest : none

The authors declare no competing interest.
